# Neurogenesis in the central olfactory pathway of adult decapod crustaceans: development of the neurogenic niche in the brains of procambarid crayfish

**DOI:** 10.1186/1749-8104-7-1

**Published:** 2012-01-06

**Authors:** Silvia Sintoni, Jeanne L Benton, Barbara S Beltz, Bill S Hansson, Steffen Harzsch

**Affiliations:** 1 Max Planck Institute for Chemical Ecology, Department of Evolutionary Neuroethology, Hans-Knöll-Straße 8, D-07745 Jena, Germany; 2 University of Bologna, Department of Biology, 40126 Bologna, Italy; 3 Neurobiology Program, Wellesley College, Wellesley, Massachusetts 02481, USA; 4 University of Greifswald, Department of Cytology and Evolutionary Biology, 17498 Greifswald, Germany

## Abstract

**Background:**

In the decapod crustacean brain, neurogenesis persists throughout the animal's life. After embryogenesis, the central olfactory pathway integrates newborn olfactory local and projection interneurons that replace old neurons or expand the existing population. In crayfish, these neurons are the descendants of precursor cells residing in a neurogenic niche. In this paper, the development of the niche was documented by monitoring proliferating cells with S-phase-specific markers combined with immunohistochemical, dye-injection and pulse-chase experiments.

**Results:**

Between the end of embryogenesis and throughout the first post-embryonic stage (POI), a defined transverse band of mitotically active cells (which we will term 'the deutocerebral proliferative system' (DPS) appears. Just prior to hatching and in parallel with the formation of the DPS, the anlagen of the niche appears, closely associated with the vasculature. When the hatchling molts to the second post-embryonic stage (POII), the DPS differentiates into the lateral (LPZ) and medial (MPZ) proliferative zones. The LPZ and MPZ are characterized by a high number of mitotically active cells from the beginning of post-embryonic life; in contrast, the developing niche contains only very few dividing cells, a characteristic that persists in the adult organism.

**Conclusions:**

Our data suggest that the LPZ and MPZ are largely responsible for the production of new neurons in the early post-embryonic stages, and that the neurogenic niche in the beginning plays a subordinate role. However, as the neuroblasts in the proliferation zones disappear during early post-embryonic life, the neuronal precursors in the niche gradually become the dominant and only mechanism for the generation of new neurons in the adult brain.

## Background

During embryonic development in the emerging ventral nerve cord of malacostracan Crustacea (for example, lobsters, crayfish and crabs), most neurons are generated by neuronal stem cells, the neuroblasts (NBs), by a division pattern called the stem cell mode [1-7]. NBs repeatedly divide asymmetrically, regenerating themselves and giving rise to one smaller ganglion mother cell that is pushed dorsally into the interior of the embryo; the ganglion mother cell divides once more to give rise to neurons or glial cells. In the crustacean brain, however, neurogenesis has not been as well documented as in the ventral nerve cord [8,9]. Evidence also suggests that the intermediate precursor cell type (ganglion mother cell) in the brain may divide more than once [10], unlike the stereotyped sequence of mitotic events described for the ventral nerve cord. It is known that in the ventral nerve cord of decapod crustaceans, NBs are mitotically active until late embryogenesis and in some cases into the post-embryonic stages, but that they cease their proliferative action before the metamorphosis to juvenile stages [2,4,10-13]. However, it is now well documented that the central olfactory pathway in the decapod crustacean brain is characterized by an addition of new olfactory interneurons that starts during early post-embryonic stages and continues throughout the life span of the animal [13-16].

Post-embryonic neurogenesis in a decapod crustacean was first shown in the spider crab *Hyas araneus *(Decapoda, Brachyura) by labeling with the S-phase cell cycle marker 5-bromo-2'-deoxyuridine (BrdU) [11]. Subsequently, studies on the adult brain demonstrated persistent neurogenesis in the central olfactory pathway of the adult shore crab, *Carcinus maenas *[17], and crayfish, *Cherax destructor *[18], where it was related to a turnover of peripheral olfactory receptor neurons on the antenna 1 [19]. Further, neurogenesis was traced from early embryonic stages through larval, juvenile and adult stages of the American lobster, *Homarus americanus*, and the long-term survival of the newborn cells was documented using pulse-chase experiments with BrdU [8]. Later studies demonstrated that the new neurons mature, differentiate and are integrated into existing circuits of the olfactory pathway [14]. To date, adult neurogenesis in the olfactory system of decapod crustaceans has been confirmed in nine species: *Cancer pagurus, C. maenas, C. destructor, H. americanus, H. araneus, Libinia emarginata, Pagurus bernhardus, Panulirus argus*, and *Sicyonia brevirostris*, suggesting that this is a common phenomenon in this group of organisms [11,13,14,16-18,20-29].

**Figure 1:**
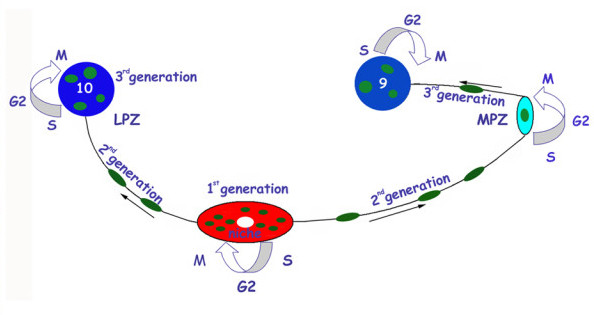
**The neurogenic system in the brain of the adult crayfish *Procambarus clarkii *(modified from **[31]). Schematic diagram of the crayfish neurogenic system in the deutocerebrum. Neuronal precursors (first generation) with glial characteristics reside within the neurogenic niche where they divide symmetrically. Their daughters (second-generation precursors) migrate along tracts formed by the fibers of the niche cells, towards either the LPZ or the MPZ. At least one more division occurs in the LPZ and MPZ before the progeny (third and successive generations) differentiate into neurons. Abbreviations: LPZ, lateral proliferative zone; MPZ, medial proliferative zone.

Adult neurogenesis in crayfish is driven by a neurogenic niche on the ventral surface of the brain in which first-generation progenitor cells reside [14,28]. This oval structure contains up to 300 bipolar cells with cytoplasmic processes that fasciculate into tracts that connect the niche with cell clusters 9 and 10. The niche also has a central cavity that is confluent with the vasculature [28,30-32]. The niche cells label with an antibody against glutamine synthetase, a marker for mammalian astrocytes and some stem cells [33-36]. Our current understanding of the functioning of the neurogenic niche in the adult crayfish brain is summarized in Figure 1. According to this model, the adult proliferative system in the crayfish brain consists of the neurogenic niche, the lateral proliferative zone (LPZ) and the medial proliferative zone (MPZ) and involves several generations of precursors that are spatially separated: the first generation neuronal precursors in the niche; their daughters (second-generation cells) that exit the niche and travel towards the proliferative zones along the migratory streams; and the third and subsequent generations, which reside in the proliferative zones to contribute progeny to the cell clusters of olfactory interneurons [14,16] (Figure 1). It should also be noted, however, that in the spiny lobster (*P. argus*), an evolutionarily less derived species than crayfish, contrasting mechanisms underlying adult neurogenesis have been proposed [13,21,29].

Despite the growing body of literature concerning adult neurogenesis in the crustacean brain, the embryonic origin of the niche is still unknown. It has been reported that the niche is already present in the second post-embryonic (POII) stage in *Procambarus clarkii *[25]. Questions related to this finding and that motivated the present study of the development of the niche are: does the niche emerge during embryogenesis or later? Does its formation coincide with that of the proliferative zones? What cells are involved in its construction and what are their origins? When does the niche initiate proliferative activity? Understanding the development of the niche and identifying the cells or tissues from which it is derived may clarify aspects of its adult function.

In this study, the development of the niche was monitored during embryonic and post-embryonic development of the brain of the procambarid parthenogenetic marbled crayfish (Marmorkrebs) [37,38]. The proliferation markers BrdU and 5-ethynyl-2'-deoxyuridine (EdU) and other immunohistochemical labels, combined with confocal laser-scan microscopy, revealed the first appearance of the niche anlagen around the time of hatching. These developmental events were confirmed in the closely related crayfish *P. clarkii*, in which additional experiments injecting fluorescently labeled dextran into the brain vasculature of embryos just prior to hatching explored the relationship between the developing niche and vasculature. The gradually changing appearance and function of the niche through development are discussed in the light of the current literature related to the adult crayfish brain.

## Results

This work describes the appearance and development of the neurogenic niche in the brain of the marbled crayfish from early stages (E38%, egg-nauplius stage) to late embryonic and post-embryonic development [39]. Images consistently show ventral views of the brain; magnifications always focus on the left hemibrain. Dextran injections into the brain vasculature of embryos just prior to hatching were done in the closely related procambarid crayfish *P. clarkii*.

### Neurogenesis in the embryonic brain is driven by asymmetrically dividing stem cells, the neuroblasts

**Figure 2:**
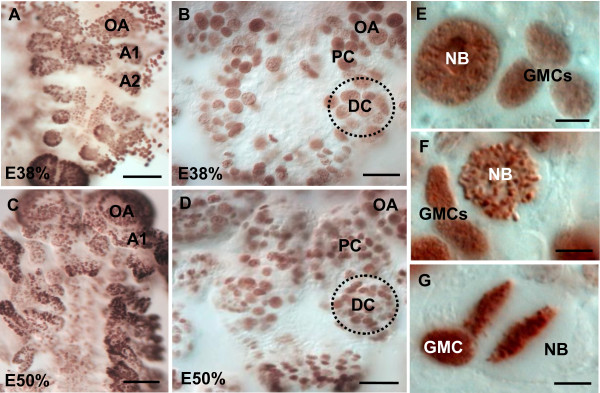
**Neurogenesis during early and mid-embryogenesis: BrdU labeling in whole mount embryos**. **(A, B) **In earlier stages (about E38%), BrdU+ cells are homogeneously distributed throughout the germ band with pronounced accumulations in the appendage anlagen. The paired deutocerebral regions show an aggregation of labeled cells (dotted line). **(C, D) **During mid-embryogenesis (E50%), the embryos are characterized by a persistently high rate of mitotic activity. The deutocerebrum (DC, dotted line in (D)) stands out from the protocerebrum and contains large spherical BrdU+ cells. **(E-G) **Higher magnification of brain neuroblasts (NBs) and associated progeny, the ganglion mother cells (GMCs). Neuroblasts can be found in early prophase (F) or in early telophase (G). Abbreviations: A1, anlage of antenna 1; A2, anlage of antenna 2; OA, optic anlagen; PC, protocerebrum. Scale bars: 50 μm (A-D), 10 μm (E-G).

**Figure 3:**
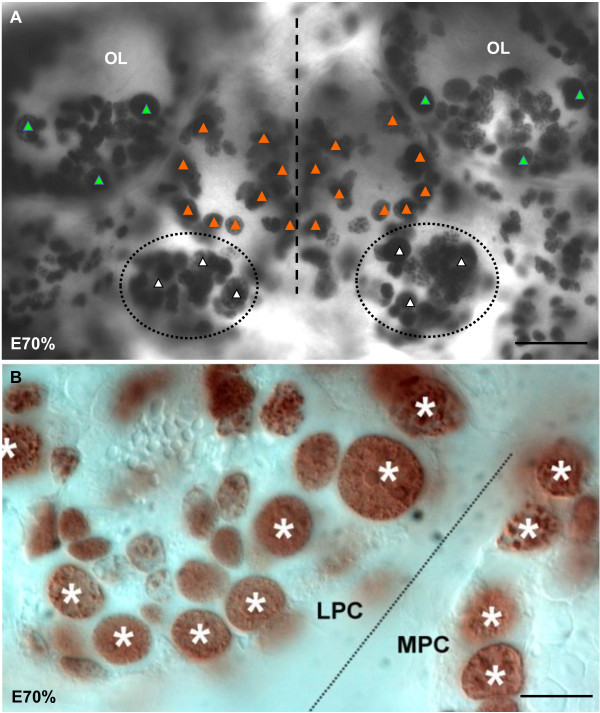
**Neurogenesis during late embryogenesis: BrdU labeling in whole mount brains**. **(A) **Arrangement of BrdU+ cells in the brain and optic anlagen of an E70% embryo. In the optic lobe anlagen (OL; and green triangles), in the protocerebrum (orange triangles) and in the deutocerebrum (white triangles) BrdU+ cells show a bilaterally symmetrical arrangement. **(B) **Higher magnification of neuroblasts (asterisks) in the medial protocerebrum (MPC) and the lateral protocerebrum (LPC; optic lobe anlagen). Scale bar: 60 μm (A), 15 μm (B).

**Figure 4:**
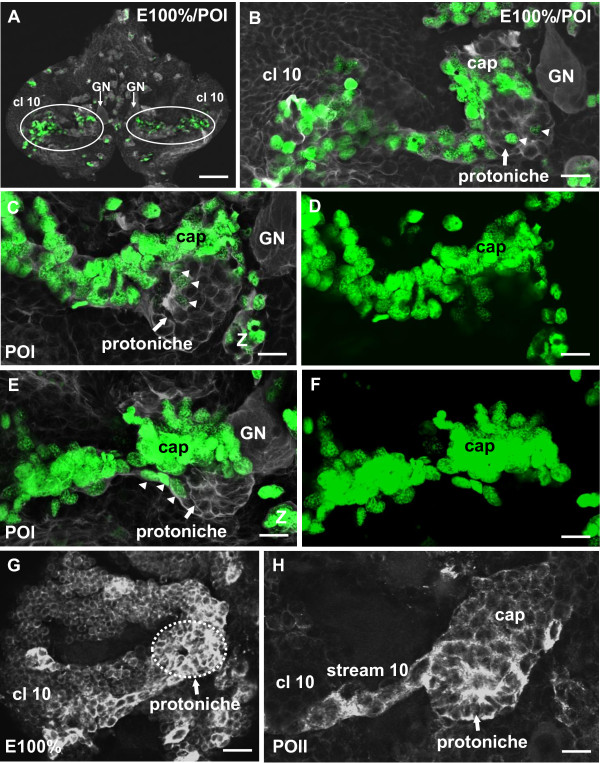
**Hatching and postembryonic stages, the emergence of the protoniche**. **(A-F) **S-phase labeling with EdU (green), and immunohistochemistry for tyrosinated tubulin (white; confocal laser-scan images). (A) Overview of the brain during hatching/POI showing the transverse deutocerebral band of mitotic cells (encircled). (B) Higher magnification of the transverse band and the forming protoniche close to the giant neuron (GN) in the left side of the brain. (C-E) Two additional specimens showing the transverse band of mitotic cells and the protoniche in the left side of the brain. Panels (D, F) show the green channels only (EdU) of (C, E), respectively. **In contrast to the numbers of dividing cells in the cap, the protoniche contains fewer and smaller EdU+ cells ( arrowheads in B, C, E)**. **(G, H) **Immunostaining for glutamine synthetase (in white), a glia cell marker, shows how the main structures of the neurogenic system (the protoniche and cap) all display immunolabeling, from the beginning of the post-embryonic life (G) to the POII stage (H) (both panels show the left side of the brain). Abbreviations: cl 10, cell cluster 10; GN, medial giant neuron. Scale bars: 100 μm (A), 20 μm (B-H).

The early development, growth and temporal sequence of ontogenic events of the marbled crayfish have been previously described [39-41]. The eggs, attached to the pleopods of the mother, contain the embryonic germ band, which resides on top of the yolk supply [39]. Around 40% of embryonic development (E40%), the first signs of the developing brain are visible when labeling with a probe for actin [41]. In the present study, *in vivo *labeling of embryos (E40% to E50%) with the S-phase-specific proliferation marker BrdU for 4 h followed by immunohistochemical processing revealed a high rate of mitotic activity in the entire germ band and especially in the anlagen of the appendages (Figure 2A, C). In the brain, BrdU-positive (BrdU+) cells are homogeneously distributed (Figure 2B, D) with the exception of the optic anlagen, where characteristic band-shaped proliferation zones merge ([9] and data not shown). At a higher magnification, large labeled nuclei belonging to the NBs (Figure 2E; asterisks in Figure 3B) and that are associated with the smaller nuclei of their progeny (the ganglion mother cells (GMCs); Figure 2E) can be distinguished (compare [2,4,8,42]). GMCs are not terminally differentiated but are precursors with the potential for additional divisions. Therefore, it is not possible to know whether GMCs labeled with a 4 h pulse of BrdU (three cells in Figure 2E) were generated by a division of the NBs during the 4-h incubation period or whether they were in S-phase themselves. After 4-h incubation, some BrdU+ NBs were found in early prophase (Figure 2F) or in early telophase (Figure 2G), demonstrating that these cells proceed from S-phase through the mitotic stages within the 4-h incubation period. We did not carry out experiments to determine the cell cycle length of the embryonic NBs, but previous studies on brain NBs in crustacean larvae suggest a cell cycle length of between 2.5 and 3 h [11,42]. During later development, NBs in some preparations are arranged in a bilaterally symmetrical pattern (triangles in Figure 3A, higher magnification in Figure 3B). By E40% and in subsequent embryonic stages, six to eight NBs and their progeny cluster in the emerging deutocerebrum (circles in Figures 2B, D and Figure 3A); these clusters transform into a bilaterally arranged transverse band of BrdU+ cells towards the end of embryogenesis (Figure 4). We will call this transient band of cells the 'deutocerebral proliferative system' (DPS) because, as we will show in the following, it gives rise to the LPZ and MPZ that we see in the adults as well as the migratory streams that connect these proliferation zones with the neurogenic niche (compare Figure 1).

### The components of the deutocerebral proliferative system become recognizable by hatching

Brains labeled with the S-phase-specific marker EdU show that neurogenesis in the brain has considerably slowed down shortly before hatching, with the exception of a few remaining NBs (for example, NB 'Z' and associated progeny in Figure 4C, E) and the DPS, which contain a mixed population of EdU+ NBs and progeny of various nuclear sizes (green channel in Figure 4A-F). The DPS extends into cell cluster 10 (Figure 4B). Labeling for tyrosinated tubulin and EdU around the time of hatching reveals that near the midline, the DPS is located just anterior to a conspicuous spherical cluster of cells, the protoniche. The protoniche also sits next to a giant neuron that, based on its location medially on the ventral surface of the brain, is presumed to be the medial giant neuron (Figure 4B, C, E) [43]. This giant neuron is a reliable landmark for the protoniche in the deutocerebrum at this stage. Taking into account the limited precision of our embryonic staging system, we determined that the protoniche appears between E90% and E100% (hatching).

In contrast to the DPS, very few cells in the protoniche incorporate EdU during a 4-h incubation period (arrowheads in Figure 4B, C, E). A strand of tubulin-rich material extends between the protoniche and the DPS; frequently, one to three BrdU+ cells in the periphery of the protoniche seem to be associated with this strand (arrowhead in Figure 4E). An antibody against glutamine synthetase, a marker of niche cells in the brain of *P. clarkii *[28], labels the cytoplasm of cells in both the DPS and the protoniche (Figure 4G, H).

### Structure of the early protoniche

**Figure 5:**
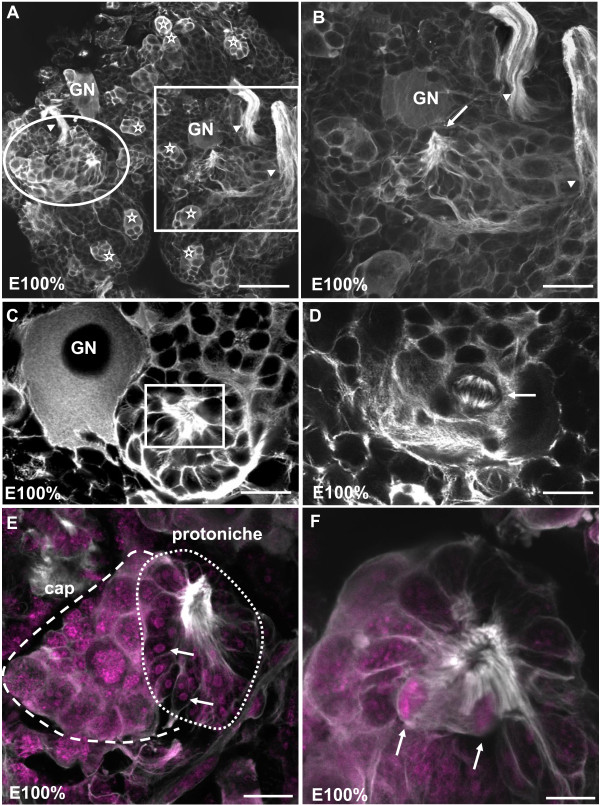
**The emergence of the niche core during hatching, immunolocalization of tyrosinated tubulin and the nuclear stain YOYO (magenta)**. **(A) **Ventral view of the brain showing two forming protoniches that are located bilaterally symmetrical on the surface of the deutocerebrum (square on the right, circle on the left) close to the medial giant neuron (GN). In the brain, clusters composed of neuroblasts and their progeny are still patchily dispersed (white stars). Arrowheads identify nerve roots. **(B) **Higher magnification of the protoniche boxed in (A). The arrow points to microtubules that converge in the center of the protoniche. **(C) **Another example of a protoniche located close to the giant neuron (GN). **(D) **Deeper focus of the specimen in (C) showing a cell in M-phase (arrow). **(E) **Within the protoniche, cells that surround the central fibrous area have very condensed, small nuclei (arrows). The cells in the cap are spherical but vary greatly in size. **(F) **Dividing cells in the protoniche close to surrounding the central fibrous area (arrows). Scale bars: 20 μm (A, B, E), 10 μm (C, D, F).

At hatching, an antibody against tyrosinated tubulin labels the embryonic nerve roots that exit the brain (arrowheads in Figure 5A, B), the medial giant neuron and compact clusters of NBs associated with their progeny (stars in Figure 5A). Tyrosinated tubulin also consistently labels the protoniche (arrow in Figure 5B, boxed area in Figure 5C) and reveals fibrous material, most likely bundles of microtubules, associated with the central cavity of the protoniche. The fibrous material is intracellular and also fills extensions of the cells in the protoniche (Figure 5E, F). In some preparations, these fibers at their point of convergence are arranged around a central cavity or pore (Figure 5F). Labeling for tyrosinated tubulin with a marker for cell nuclei (YOYO) reveals that the cell nuclei in the protoniche are small and condensed (arrows in Figure 5E, F) when compared to the surrounding brain tissue. Optical sections deeper into the protoniche reveal the presence of cells in anaphase that appear to be undergoing geometrically symmetrical divisions (arrow in Figure 5D). Furthermore, the protoniche is associated with a compact cell cluster (the 'cap') with spherical nuclei of various sizes that are much larger than the nuclei in the protoniche itself (Figure 5E).

### In the POI stage the DPS differentiates into the cap and LPZ, which are linked by a stream of cells in S-phase

**Figure 6:**
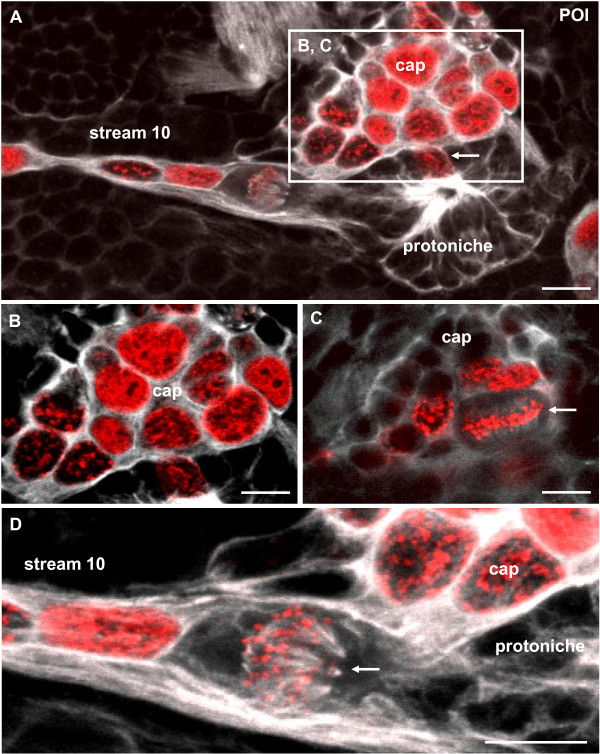
**The protoniche and cap in the POI stage, left side of the brain**. Double labeling for the S-phase marker BrdU (red, 4-h pulse) and for tyrosinated tubulin (white). **(A) **Overview of the protoniche and stream 10. The arrow identifies a cell in S-phase. The boxed area is magnified in (B, C). **(B) **Within the cap, a size continuum of BrdU-labeled cells is present. **(C) **Deeper focus of the cap showing a cell that divides in a geometrically asymmetric way (arrow). **(D) **Higher magnification of the stream 10 as it emerges from the protoniche. The arrow identifies a cell in M-phase. Scale bars: 20 μm (A), 10 μm (B-D).

BrdU and tyrosinated tubulin labeling reveal that by POI the DPS has differentiated into the LPZ associated with cell cluster 10 and the cap that sits antero-medially to the protoniche (Figures 4C, E and 6A). The LPZ and cap are linked by a thin bundle of tubulin-rich filaments that are associated with BrdU+ cell nuclei that have an elongate shape. We identify this bundle of filaments as the migratory stream that links the LPZ and the neurogenic niche in adult crayfish (see schemes in Figure 1) and will refer to this bundle as 'stream 10'. After a 4-h exposure to BrdU, the cells that make up the protoniche are only rarely labeled (Figure 6A), while almost all nuclei in the cap are BrdU+. The BrdU+ nuclei in the cap are of a variety of sizes, making it impossible to distinguish which of these cells are NBs and which are intermediate (second generation) precursors (Figure 6B). Optical sections taken deeper into the cap reveal metaphase cells that seem to be undergoing geometrically symmetrical divisions (arrow in Figure 6C). Furthermore, mitotic cells are frequently encountered at the position where stream 10 is linked to the protoniche (for example, Figure 6D), and these also appear to divide in a geometrically symmetrical pattern.

### Neuroblast-X and the cap contribute progeny to cluster 9 in POI and POII stages

**Figure 7:**
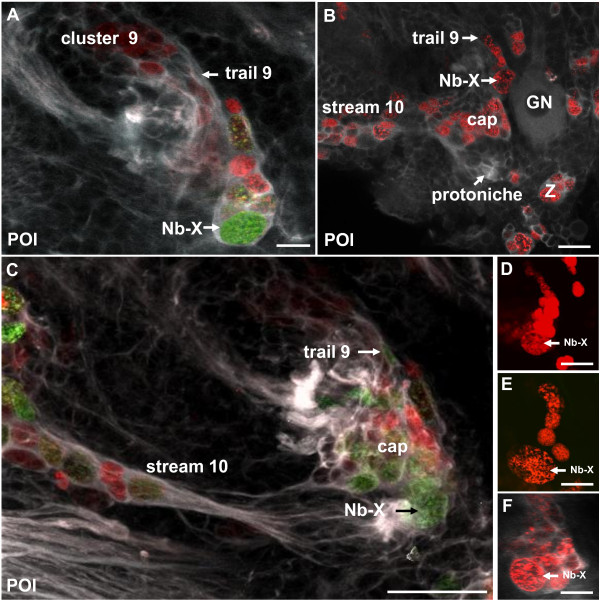
**Neuroblast X, the cap and trail 9 (left side of the brain) in double-pulse experiments (48-h interval between the pulses) with the S-phase markers BrdU (first pulse, red) and EdU (second pulse, green) combined with immunolocalization of tyrosinated tubulin (white); late POI stages**. **(A) **Neuroblast X (NB-X) and its progeny are embedded in tubulin immunoreactive material. A string of progeny extends towards trail 9. **(B) **Overview of the cap, NB-X, stream 10 and trail 9 close to the medial giant neuron (GN). Neuroblast Z is also shown as a landmark (Z). **(C) **The same specimen as in (A) but in a more superficial focus plane showing close association of the cap and NB-X, which both contribute progeny to trail 9. **(D-F) **Additional specimens showing NB-X and the associated trail of progeny towards cluster 9 (only the red channel of the first pulse is shown and tyrosinated tubulin in (F)). Scale bars: 10 μm (A), 20 μm (B, D), 50 μm (C), 10 μm (E, F).

**Figure 8:**
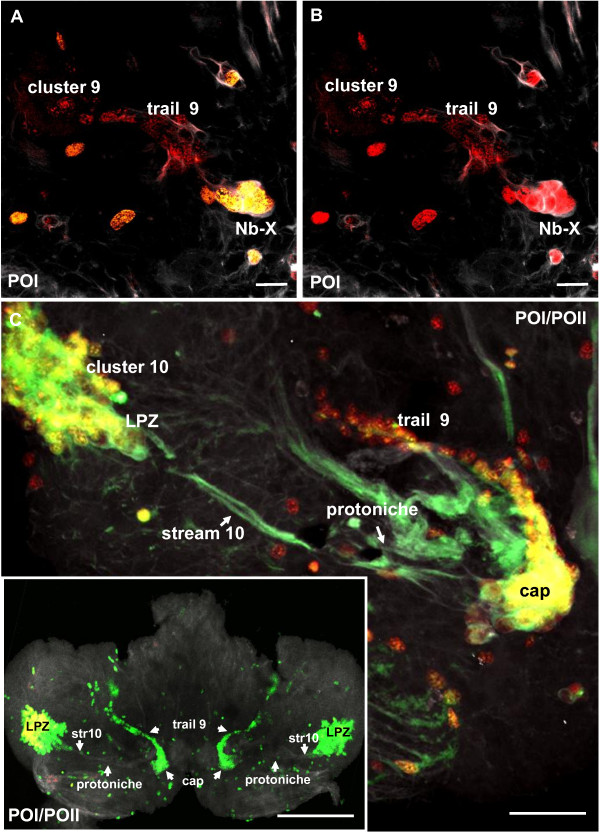
**Neuroblast X, the cap and trail 9 (left side of the brain) in double-pulse experiments (48-h interval between the pulses) with the S-phase markers BrdU (first pulse, red) and EdU (second pulse, green) combined with immunolocalization of tyrosinated tubulin (white); late POI and early POII stages**. **(A, B) **Both panels show the same specimen but (B) shows only the red label for the first pulse. Labeled cells in trail 9 and cluster 9 have only incorporated the first label and are not cycling anymore during the second pulse. **(C) **This specimen was a POI during the first pulse and hatched to POII during the 48-h chase period before the second pulse. The cap is already transforming into the medial proliferation zone (MPZ; compare Figure 14). The cells in trail 9 show only the first label (red) whereas most cells in the lateral proliferation zone (LPZ) and MPZ show both labels and therefore appear yellow. This suggests that cells in trail 9 may be post-mitotic and on their way to differentiation in cluster 9. Note strong crosstalk of the white channel (tubulin) and the green channel so that stream 10 and several nerve roots appear green instead of white. Stream 10 (str10) is devoid of any mitotic cells. The inset in (C) shows an overview of the ventral side of a brain in a further advanced POII stage showing the arrangement of the LPZ, and trail 9. Scale bars: 20 μm (A, B), 50 μm (C), 200 μm (C inset).

In POI crayfish, one large BrdU/EdU+ cell in the DPS displays features of a typical embryonic NB and is here named NB-X (Figure 7). NB-X was frequently encountered close to the landmark giant neuron in optical sections that were more dorsal and anterior than those that revealed the cap, which sits on the ventral surface of the brain (Figure 7A, B). Tyrosinated tubulin labeling (Figure 7A, F) revealed that NB-X and some of its progeny are embedded in a common matrix of fibrous material, additional evidence for the clonal relationship between these cells. The progeny of NB-X are lined up like pearls on a string that extend anteriorly and then laterally towards cell cluster 9 (Figure 7B-F), which in the adult brain houses the somata of local olfactory interneurons. We use the term 'trail 9' for this string of cells between NB-X and cluster 9 (Figure 7A-C); the cap also appears to contribute progeny to trail 9 (Figures 7A and 8C). Pulse-chase labeling experiments where a 4-h pulse of BrdU was followed after 48 h by a 4-h pulse of EdU more clearly revealed trail 9 (Figure 8A-C; inset, Figure 8C); in contrast, the EdU label was only present in NB-X and its immediate progeny (Figure 8A).

These studies suggest that during the 48-h chase period, at least some of the progeny of NB-X and the cap migrate to or become displaced towards cell cluster 9. Because we do not know the BrdU clearing time, it is possible that some of the BrdU+ cells in trail 9 were labeled only shortly before the EdU pulse. In fact, experiments in brains of embryonic lobsters (*H. americanus) *[10] and the crayfish (*P. clarkii*) [30] suggest that the clearing time of BrdU in these organisms is approximately 2 days. In POII animals, some NBs are still present in parts of the brain other than the DPS (data not shown), but NB-X is no longer visible. Whether the NB-X has ceased its proliferative action and died or has been incorporated into the cap is not known.

### POI pulse-chase experiments: the cap, protoniche and LPZ

**Figure 9:**
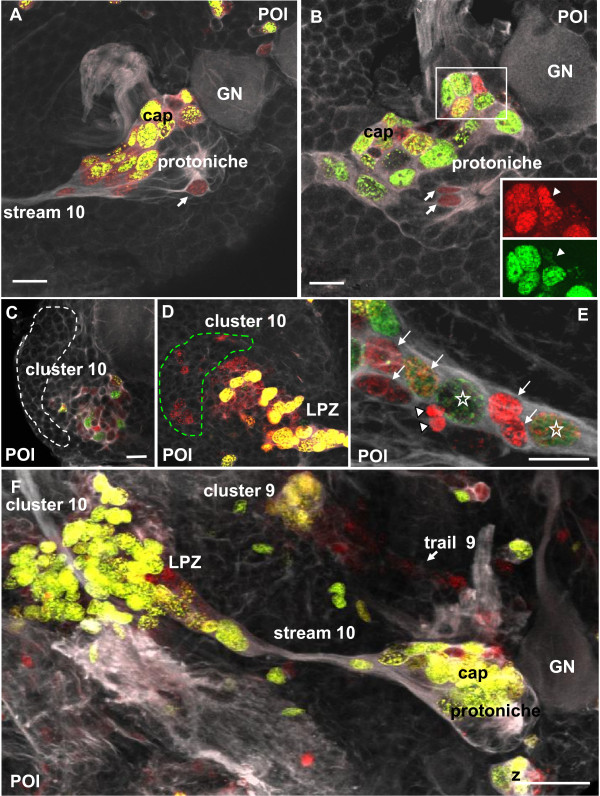
**The deutocerebral proliferative system in double pulse experiments (48-h interval between the pulses) with the S-phase markers BrdU (first pulse, red) and EdU (second pulse, green) combined with immunolocalization of tyrosinated tubulin (white); POI stage**. **(A) **In the cap most cycling cells have incorporated both labels. One exception is shown in the inset in (B), where one cell only has incorporated the first label (arrowheads in the inset). Note that compared to the cap, very few cells are in S-phase in the core (arrows **in A and B**). **(C, D) **Cluster 10 (dotted line) and lateral proliferation zone (LPZ) are shown. A mix of dividing cells and progeny is present. Panel **(**D**) **shows the same specimen as (C) but is focused deeper into the tissue. The green dashed line encircles a zone in which only cells with the first label are present. These may be postmitotic cells that are on their way to differentiation and being integrated into cluster 10. **(E) **Stars identify two large nuclei that have incorporated both labels and hence were actively cycling during the second pulse. The white arrows identify smaller cells. Most of these have only the first label but one of these intermediate cells has both labels. The white arrowheads identify two cells that may represent two telophases. **(F) **Overview of the deutocerebral proliferative system. Note the strong mitotic activity in the LPZ and the cap. Abbreviations: LPZ, lateral proliferative zone; GN, medial giant neuron. Scale bars: **20 μm (A, B, C), 10 μm (E); 50 μm (F)**..

Pulse-chase experiments (first pulse BrdU, second pulse EdU after 48 h) demonstrated that, in the POI stage, most cells in the cap are in S-phase (Figure 9A, B); both labels were incorporated into many of these cells, which may be due to their persistent cycling and/or the possibility that BrdU is available during most of the 48-h chase period. In contrast, few cells in the protoniche label (one to two per labeling period; Figure 9A, B), suggesting that the cell cycle rate is very slow compared to cells in the cap. Second, cells in the protoniche generally label with only one marker, indicating that the S-phase is shorter than the clearing time (48 h). Pairs of labeled cells are frequently seen in the protoniche, and it is unclear in these cases (for example, Figure 9A, B) whether these are siblings or if they entered S-phase independently.

These experiments also revealed intense mitotic activity in cells in the LPZ that extends into cell cluster 10 (Figures 8C and 9C, D, F); many of these incorporated both labels whereas others incorporated only one (Figure 9B, E). Those cells that incorporated only BrdU (and hence were not in S-phase during the second pulse) typically were arranged laterally towards cell cluster 10 and away from where stream 10 enters the LPZ (Figure 9C). These data suggest that these cells have exited the cell cycle prior to the EdU pulse and may be differentiating into neurons. The LPZ proximal to the entry of stream 10 typically housed cells that incorporated both labels and were larger, suggesting that these may be progenitors that were still cycling during the second pulse and therefore may undergo multiple divisions (Figure 9E). Unfortunately, we did not encounter any M-phase cells at this site in our specimens so we do not know if these cells divide asymmetrically or symmetrically; however, the position of some paired cells (for example, Figure 9E) suggests that symmetric divisions do occur in stream 10 as in adult crayfish [32]. The multiple cell sizes also indicate the potential for multiple generations of precursor cells in the LPZ.

**Figure 10:**
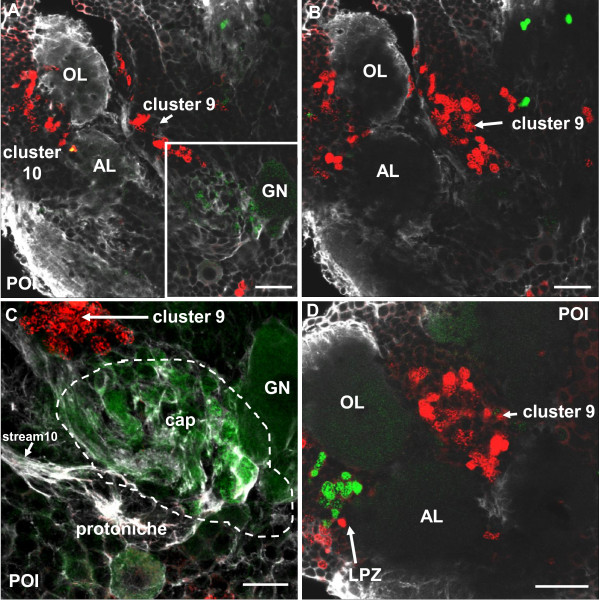
**The deutocerebral proliferative system and protoniche (left side of the brain) in double-pulse experiments (7-day interval between the pulses) with the S-phase markers BrdU (first pulse, red) and EdU (second pulse, green) combined with immunolocalization of tyrosinated tubulin (white); animals were in POI stage for the first pulse and in the early POII stage for the second pulse**. **(A, B) **Different focal planes of the same specimen. After 7 days, all cells labeled in the first pulse have moved out into clusters 10 and 9. The green cells in the upper right corner may be glia cells. The boxed area in (A) is magnified in (C). **(C) **Higher magnification of the protoniche. Note that the green fluorescence in the cytoplasm of the medial giant neuron (GN) most likely is background labeling. **(D) **Higher magnification of a deeper focal plane of the same specimen as in (A-C) reveals that the lateral proliferation zone (LPZ) is mitotically active in the second pulse (green). Unspecific background visualizes the accessory lobe (AL) and olfactory lobe (OL). Scale bars: 50 μm (A, B, D), 20 μm (C).

Pulse-chase experiments with a 7-day interval between the two pulses (the animals had molted to the POII stage during this time) revealed that after one week the mitotic cells that incorporated the first label had completely moved out into cell clusters 9 and 10 (Figure 10A, B). Stream 10 was devoid of labeled cells in these specimens (Figure 10C). The second label revealed continuous proliferative activity in the cap (Figure 10C) and the LPZ (Figure 10D).

### POII stage and juveniles (14 mm body length): detachment and displacement of the cap

**Figure 11:**
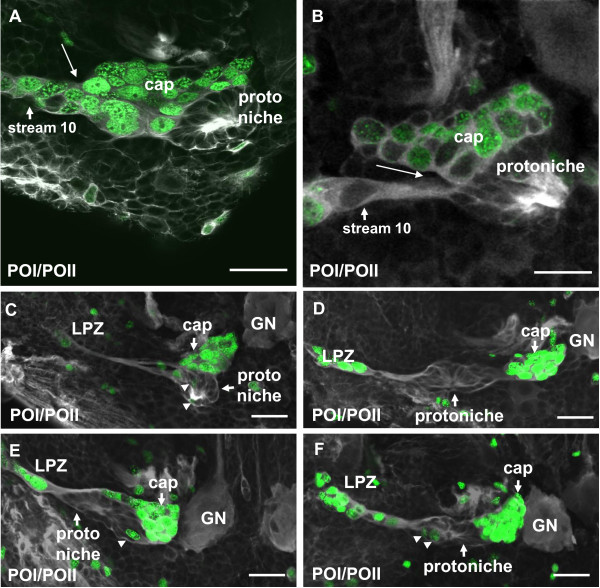
**Separation of cap and prototoniche during the transition from late POI to early POII, immunolocalization of tyrosinated tubulin (white) and single 4-h pulse of the S-phase marker EdU (green)**. **(A, B) **The cap starts to detach from the protoniche (white arrow). **(C-F) **Four different specimens showing various stages of the displacement of the cap towards cluster 9. In our terminology, once the cap has completely detached from the core, the latter is called the neurogenic niche as in adults (Sullivan et al., 2007a, b). The cap becomes the medial proliferative zone. **Note that compared to the cap, very few cells are in S-phase in the protoniche (arrowheads in C, E, F)**. Abbreviations: GN, giant neuron; LPZ, lateral proliferative zone. Scale bars: 20 μm (A, B); 30 μm (C-F).

**Figure 12:**
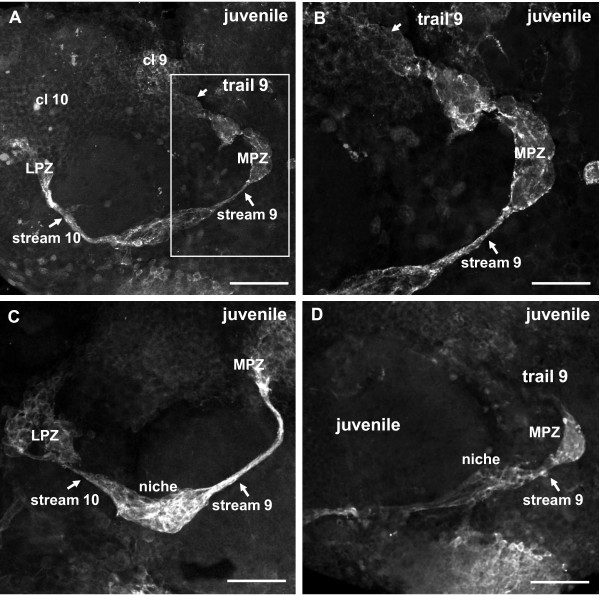
**Late POII/early juvenile (ADI, 14 mm carapace length): the neurogenic niche and proliferation zones stained for gluthamine synthetase**. **(A, D) **Displacement of the medial proliferation zone (MPZ; former cap) towards cluster 9 in three different specimens. **(B) **A higher magnification of the boxed area in (A). **(C) **In this juvenile specimen, the MPZ (former cap) has already relocated from the niche and reached cluster 9. This arrangement is now very similar to that in adults [14,28] (Figure 1). Abbreviations: CL 10, cluster 10; LPZ, lateral proliferative zone; MPZ, medial proliferation zone. Scale bar: 100 μm (A, C, D), 50 μm (B).

While the animals undergo a molt to the POII stage, the DPS is transformed as the cap becomes more and more distinct from the protoniche and starts to detach from it (Figure 11A-F). At this stage stream 10 clearly emerges from the protoniche and is not connected to the cap (Figure 11B, C). As the cap and protoniche are displaced, the protoniche shifts laterally towards the LPZ and the cap moves medially towards the medial giant neuron (Figure 11C-F). As in the POI stage, very few of the protoniche cells incorporate the S-phase marker (arrowheads in Figure 11C, E, F), in contrast to the abundance of label in the cap. In juvenile animals, labeling with glutamine synthetase reveals that the cap and protoniche are further displaced (Figure 12) and finally achieve an arrangement that is more or less similar to the situation described for adult crayfish (Figure 1). The cap is now located closer to cluster 9 and in a position where in adult animals the same labeling technique has shown that the MPZ is located (Figure 12) [14]. A thin strand of glutamine synthetase-positive material links the protoniche and cap. We suggest this strand is equivalent to the migratory stream, which in adults links the neurogenic niche and the MPZ; this strand is referred to as 'stream 9'. Hence, the embryonic protoniche in the crayfish brain develops into what in adults has been named the neurogenic niche; the embryonic cap develops into the MPZ (Figure 1) [28].

### Injection of fluorescently labeled dextran into the dorsal hemolymph sinus of embryos and hatchlings reveals a close association between the vasculature and the protoniche

**Figure 13:**
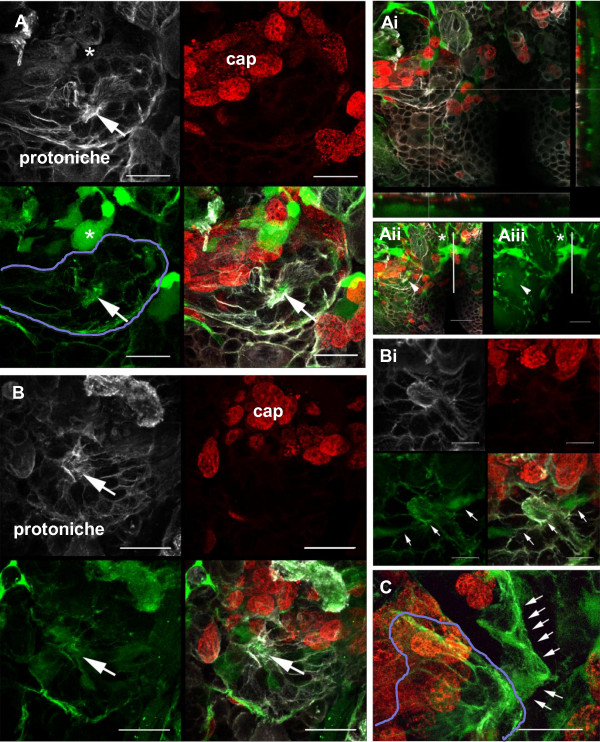
**Dextran dye micro-injections (green) into the cerebral artery via the dorsal sinus of embryos at E95% to E100%/POI stage**. The S-phase marker BrdU (red) identifies the cap and the immunolocalization of tyrosinated tubulin (white) delineates the protoniche. Panels (A, Ai, Aii, Aiii) show the same specimen. **(A) **All three channels are shown separately with the composite image at the bottom right: top left, tyrosinated tubulin (white); top right, Brd U-labeled cells (red); bottom left, dextran dye filled blood vessels (green) running through the protoniche (outlined in blue). An example of a lacunae filled with dextran dye (asterisk), a capillary that has an expanded blind ending, is seen in the bottom left of (A), and an asterisk is also placed where it exists in the tyrosinated tubulin channel, top left of (A). **(Ai) **Leica software for the orthogonal slicer function was applied to this stack of images to obtain coronal (xz-axis) and sagital views (yz-axis) through the pore and the protoniche at the dotted reference lines. The intersection of the reference lines marks the center of the pore, which can be seen in the z-axis at the bottom and right side, showing the vasculature running concommitant with the pore and just underneath the pore and the protoniche. **(Aii) **A confocal stack (26 μm) from the ventral surface through to the mid-brain in a compressed view, showing that the cerebral artery (asterisk) bifurcates and then pushes ventrally toward the two hemispheres, each branch dividing laterally and then many times over into the neuropile regions as continuous tubes. The solid vertical line demarks the midline of the brain and the arrowhead denotes where the pore of the niche is located. **(Aiii) **The dextran (green) channel alone, more clearly illustrating the vasculature pathway from the midbrain (via the cerebral artery) to the ventral regions of the brain, and finally to regions around and in the niche. **(B) **Another example of a protoniche that has blood vessels confluent with the niche structure. The separate confocal channels are the same as in (A), and the arrow points to the central fibrous area of the pore. **(Bi) **A slightly higher magnification of (B) imaged deeper into the pore, showing the vasculature (arrows) running through the pore itself. All three channels are displayed as described for (A, B). **(C) **The vasculature can sometimes be observed to be contiguous with the protoniche (outlined in blue). The arrows point to a blood vessel that becomes part of the protoniche structure. Scale bars: 20 μm (A, Aii, Aiii, B, C), 10 μm (Bi).

It has been reported that the neurogenic niche in adult crayfish is confluent with the vasculature via a 'vascular cavity' located centrally within the niche. This relationship has been demonstrated by injection of fluorescently labeled dextran into the dorsal artery of the brain (*P. clarkii *[28]; *C. destructor *in [44]) or into the pericardial sinus (*P. clarkii *[30]). Therefore, to explore the relationship between the developing niche and the vascular system, fluorescently labeled dextran was micro-injected into the dorsal sinus in embryos of *P. clarkii *just prior to hatching (Figure 13; Additional file 1). These injections resulted in dye distributed throughout the brain vasculature, including a network of capillaries previously described by Sandeman [45] in the crab *C. maenas*; the vascular system in the brain of *P. clarkii *is the same in all major respects [28]. In adults, the cerebral artery, which enters the brain medially on the dorsal surface, plunges down through the deutocerebrum, giving off a medial anterior branch to the protocerebral neuropils. When the artery reaches the ventral side of the brain it divides into two large lateral branches that go to the accessory and olfactory lobes, and antenna 2 neuropils.

Dye injections into the dorsal sinus of embryos revealed the cerebral artery and its lateral branches serving the accessory and olfactory lobes. Primary branches of the cerebral artery were as previously described for the adult crab brain [45], as were the capillary systems that emanate from the arteries. As in the adult crab brain, the capillaries in the embryonic crayfish brain run as continuous tubes with many junctions and endings where they expand into what Sandeman termed 'lacunae' [45]. Such lacunae can be visualized in the region of the protoniche (Additional file 1). As in adults, the capillary systems in the embryonic brain correspond well with the neuropil regions. Thus, our injections appear to have thoroughly filled the vasculature in the brain, revealing not only major arteries but also the finer structure of the capillary systems (Additional file 1).

The dextran injections were combined with BrdU and and tyrosinated tubulin labeling in E95% to 100% embryos and in POI stage *P. clarkii*. BrdU labeling identifies the DPS and cap, while the immunolocalization of tyrosinated tubulin delineates the protoniche (Figure 13A, B); these markers show that the spatial relationships between these structures at this time in development are identical in *P. clarkii *to what was observed in it closest relative, the marbled crayfish, described above. Further, the dextran injections demonstrate that from its first appearance in embryos just prior to hatching, the protoniche is closely associated with the vasculature. A fine capillary that emerges from the lateral ventral branches of the cerebral artery is intertwined with the protoniche, with extensions inserting into and running throughout the region (Figure 13Aii, Aiii, C; Additional file 1). Dextran also is readily visualized within the central fibrous area of the protoniche, which in the adult becomes the vascular cavity (Figure 13A, B, Bi composite images; Additional file 1); this demonstrates that the vasculature communicates directly with the niche, even at the earliest stage at which the protoniche can be identified. These features of the niche and vasculature are highly stereotyped, and were observed repeatedly in every embryonic brain examined.

It is notable that the pattern of labeling for tyrosinated tubulin corresponds closely to the dextran labeling of vascular elements. This is not due to bleed-through between the emission channels. This is readily confirmed by examining dextran labeling of lacunae (Figure 13A bottom left, asterisk), for instance, where there is no labeled structure in the tyrosinated tubulin emission channel, suggesting that there are either no microtubules in these structures or that the tubulin is not tyrosinated (Figure 13A top left). The dextran/tyrosinated tubulin double labeling of the fibrous elements that form the core of the protoniche therefore suggests that the first developmental appearance of the niche is closely aligned with, or possibly part of, the developing vasculature.

## Discussion

In the current project, a model for the development of the neurogenic niche in the procambarid crayfish brain is proposed. The DPS emerges on the ventral side of the brain around the time of hatching, and is found adjacent to a new structure, the protoniche. As the last deutocerebral NBs disappear during post-embryonic stages, the protoniche gradually differentiates and becomes the dominant and only source of neuronal precursors in the adult crayfish brain. The origins of cell clusters 9 and 10 and the migratory streams have been identified, but the cellular origins of the protoniche cells remain obscure. These findings contribute to our understanding of neurogenesis in the juvenile and adult stages of the closely related crayfish *P. clarkii *[13,14,16,25,27,28,30,31,46,47].

### Emergence of the deutocerebral proliferative system

To our knowledge the only available study on the development of a crustacean neurogenic niche is that of Song *et al*. [25] on the crayfish *P. clarkii*. These authors suggested that the 'neurogenic complex' was already in place when the embryos hatch and proposed that 'it must be generated during embryonic development ... as a compact structure that becomes elongated in adults' [25]. However, these authors did not provide any data on late embryogenesis to support this assumption. Furthermore, both POI and POII stages were grouped together in their study and defined as 'post-embryonic hatchlings' and, in particular, the POII stage of *P. clarkii *apparently was mistaken for the POI (animals still connected to the mother by anal thread) [48]. Therefore, the period defined in the current study during which the emergence of the neurogenic niche, streams, LPZ and MPZ occurs has not been examined in a consistent and controlled fashion.

**Figure 14:**
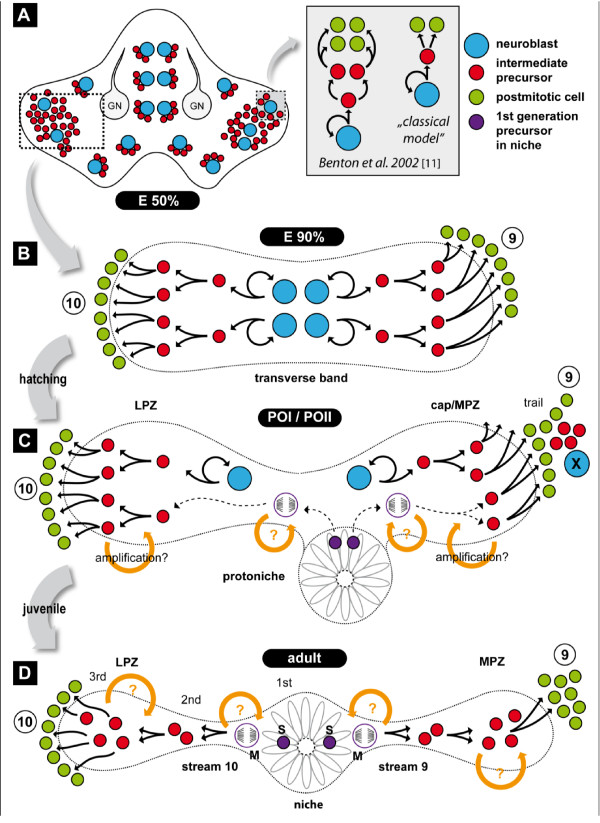
**A model for the development of the niche in the marbled crayfish brain**. **(A) **Overview of the embryonic brain just before hatching. Neuroblasts (NBs) (blue circle) and their progeny (red circle) are present and patchily dispersed in the brain. The boxed area shows two alternatives conerning neuroblast division in the embryonic brain. **(B) **Magnification of the rectangular area outlined in A) To show the transverse band of cells at E90%. **(C) **The protoniche and deutocerebral proliferative system at POI and POII stages. (D) The Deutocerebral proliferative system in the adult crayfish brain. Abbreviations: 1st, 2nd, and 3rd, first, second, and third generation progenitors; 9, cell cluster 9 (local olfactory interneurons); 10, cell cluster 10 (olfactory projection neurons); GN, medial giant neuron; LPZ, lateral proliferation zone; M, cell in M-phase; MPZ, medial proliferation zone; POI, post-embryonic stage I; POII, post-embryonic stage II; S, cell in S-phase; X, neuroblast X.

It is well known that asymmetrically dividing stem cells, the NBs, drive embryonic neurogenesis not only in the ventral nerve cord [7] but also in the brain [8,9] of malacostracan crustaceans. As in other crayfish species [49], we encountered such NBs in the brain of the marbled crayfish during early phases of embryogenesis. Towards late embryogenesis, in addition to the single, patchily dispersed NBs, bilateral compact transverse bands of BrdU-positive cells form in the developing ventral cell cortex of the deutocerebrum. Such bands of mitotic cells previously were reported in the deutocerebrum of lobster embryos [8] and in the brains of crab embryos and larvae (Figure 8A in [42]). In the present study, we show that this transverse band of cells, which we termed the deutocerebral proliferative system, gives rise to the MPZ and LPZ as well as the migratory streams that link these structures. These elements of the DPS and the protoniche develop according to a specific sequence of events that are characteristic of specific stages (Figure 14): late embryogenesis, hatching, the POI and POII stages, until the early juvenile (or ADI, the first immature adult stage as defined in [48]). Our data suggest that the formation of the DPS takes only a relatively short period of time.

The DPS comprises an array of NBs, ganglion mother cells and/or other intermediate precursors. The term NB in arthropods classically refers to neuronal stem cells that divide in a geometrical and molecularly asymmetrical pattern and give rise to ganglion mother cells (reviewed in [50,51]). Recent studies in insects have proposed an additional type of mitotically active cell besides NBs and ganglion mother cells, the secondary amplifying progenitors that originate from a certain subtype of ganglion mother cell and contribute to specific neuronal lineages [52,53]. Such progenitors have been found in *Drosophila melanogaster *to contribute to post-embryonic protocerebral structures [52,54-56] and are also present in the embryonic brain of *Schistocerca gregaria *[51]. In grasshopper, these so-called intermediate or amplifying progenitors are responsible for a novel mode of neurogenesis that amplifies a large lineage of cells in a short period of time. These new findings expand our knowledge on neurogenesis of insects, in which ontogeny, number, cell lineage, fate of NBs, as well as other proliferative cell types have been identified by both molecular and morphological means [51,57-60].

The mitotic cells of the DPS not only comprise NBs but also a heterogeneous assortment of intermediate-sized cells that are positive to S-phase markers and seem to be involved in the post-embryonic rapid massive production of cells in the deutocerebrum. They may be functionally equivalent to the insect intermediate or amplifying progenitors and the presence of such a cell type has already been suggested in the literature on crustacean post-embryonic neurogenesis in the brain [16,30,61]. However, more information on cleavage plane, asymmetry of division, expression of specific molecular markers, and also positional information is needed to clarify their identity.

During late embryogenesis and early post-embryogenesis, the DPS divides into two parts, laterally into the LPZ and medially into the cap. Both the LPZ and the cap contain numerous cells in S-phase at this stage. At the same time, an additional structure, the protoniche, which will become the neurogenic niche of the adult crayfish [14,28], emerges just posterior to the cap (Figure 14C). In contrast to the cap, where the majority of cells are in the cell cycle at any given time, there are only few proliferating cells in the protoniche. Further, the cap and the protoniche have clearly diverging anatomical features: the protoniche looks like a radial corona of spindle-shaped cells that have extensions containing tubulin labeling that converge on a central pore; in contrast, the cap is a hub of cells with spherical nuclei, and S- and M-phase nuclei are abundant. It remains unclear what kind of interactions may occur between the adjacent cap and protoniche. However, what emerges from our data is that the protoniche and the LPZ are connected very early in post-embryogenesis by the forming migratory stream 10. The LPZ, stream 10 and protoniche are all immunopositive for glutamine synthetase, suggesting some kind of early relationship among these structures.

The structure that in adults is known as the MPZ derives from the cap during POII/ADI, when the cap and protoniche are displaced away from each other. Our data demonstrate that while the migratory stream 10 between the protoniche and LPZ is present at hatching and gets thinner and more defined while the brain matures, the stream between the protoniche and MPZ emerges only after the detachment of the cap from the protoniche (POII-juvenile). Therefore, the components of this neurogenic system do not form simultaneously and may not derive from the same cell source (that is, there are no data supporting a derivation of the protoniche from the DPS), but the development of the system embraces a period of time that lasts about two weeks (from hatching until ADI).

### Relationship of the post-embryonic deutocerebral proliferative system and the adult neurogenic niche

Our study suggests that persistent neurogenesis in the early post-embryonic stages is mostly driven by local amplification in the LPZ and MPZ, while the protoniche in the beginning is characterized by a very low level of mitotic activity and plays a subordinate role. Hence it would appear that during early post-embryogenesis the proliferation zones operate relatively independently from progeny that reach them from the protoniche via the migratory streams. The proliferative characteristics of the protoniche (low level of mitotic activity) closely resemble those in the adult crayfish niche [28,30]. The origin of first-generation precursors in the adult neurogenic niche is currently under investigation and a new hypothesis has been proposed [30]. The first-generation precursors in the crayfish *P. clarkii *undergo geometrically symmetrical divisions and both daughter cells migrate away [32], suggesting that these divisions are not self-renewing. This has been confirmed in experiments that directly tested the self-renewal capacity of the niche cell population [30]. Hence, the first-generation neuronal precursors are not typical neuronal stem cells. These data also imply that the niche precursors must come from a source extrinsic to the niche. Recent *in vitro *studies have shown that cells circulating in the blood (hemocytes) are attracted to and incorporated into the niche, suggesting that the hematopoietic system may play a role in maintaining the supply of neuronal precursors in the niche [30].

Our studies in the marbled crayfish identified the timing of appearance of the niche anlagen and followed the differentiation of the neurogenic system during post-embryonic stages. Additional work in the crayfish *P. clarkii *extended this understanding by confirming features of the DPS and protoniche just prior to hatching and in early post-embryonic stages. Further, these studies explored the relationship between the vascular system and the protoniche. The vascular system appears to be a common feature of all stem cell niches, including those in the nervous system [62,63]. In both the subventricular and subgranular zones, which support adult neurogenesis of olfactory bulb and hippocampal neurons, respectively, the stem cells lie in close proximity to a rich plexus of blood vessels that are conduits for circulating hormones and cytokines released from distant sources [64]. Endothelial cells, which are known to regulate stem cell self-renewal and neurogenesis via a cadre of secreted factors, are emerging as critical components of neurogenic niches [65-67]. In addition, the extracellular matrix associated with the vasculature appears to provide a means of cell anchoring in the niche [66]. The vascular elements in niches therefore create a microenvironment and architecture that support stem cell division and provide directional cues for migration of their descendants [63,67,68].

The literature on 'neurovascular' relationships is rapidly expanding, particularly as these relate to mechanisms of adult neurogenesis, although the role the vascular system may play in the development of neurogenic niches is not known. It is interesting, however, that the subgranular zone was first defined in mammals as the co-association of newborn neurons with dividing endothelial cells [69]. Further, work in the adult songbird brain suggests a strong and dynamic association between angiogenesis and neurogenesis [70]. Testosterone treatment of adult female canaries can elicit primitive song in these otherwise songless birds, an effect that has been attributed to a direct causal interaction between testosterone-induced angiogenesis and neurogenesis. Following on these findings, Shen et al. (2004) demonstrated in vitro that endothelial cells release soluble factors that stimulate neural stem cell division and enhance neuronal production [65]. An extensive list of neurovascular interactions that influence neural stem cells have now been defined, thus establishing that niche function in the vertebrate brain is dependent on a close relationships with the vasculature [63]

Communication between the blood vasculature and the crustacean neurogenic niche has been demonstrated previously [28,30,44], although molecular aspects of this relationship are not yet known. The present study demonstrates that the first developmental appearance of the neurogenic niche in crayfish is temporally and spatially associated with the formation of local vascular elements. While causal interactions between the forming capillaries and the niche cannot be defined based on our current experiments, the apparent association between angiogenesis and neurogenesis in the developing crustacean brain exhibits remarkable parallels with findings in vertebrates. Our data therefore suggest highly conserved mechanisms underlying adult neurogenesis in vertebrate and invertebrate species, as proposed previously [28]. In addition, these findings are suggestive that the assembly of the crustacean neurogenic niche may be coupled with angiogenesis.

## Conclusions

This study defines the appearance of proliferative areas during early post-embryonic stages that will persist and become the functional elements producing neurons throughout the life of the organism. The protoniche, which originates in close association with vascular elements, is first identifiable at around the time of hatching. Although protoniche cells are actively in the cell cycle, they proceed very slowly compared with other proliferative structures in the post-embryonic brain, a feature that is also indicative of niche function in the mature brain. In contrast, the LPZ, which arises in the POI stage from the DPS, contains cells that are rapidly proliferating. The MPZ does not become recognizable in its adult form until POII, when the post-embryonic cap, derived from the DPS, separates from the protoniche. As the brain expands during the early juvenile stages (ADI), the migratory streams linking the protoniche to both the MPZ and LPZ extend. During POI and POII, the streams do not readily label with S-phase markers, suggesting that they do not yet contain cells migrating between the niche and the proliferation zones (LPZ and MPZ); this is consistent with the slow cycling activity of cells in the protoniche. Our data therefore suggest that the LPZ and MPZ are largely responsible for the production of new neurons in the early post-embryonic stages, and that the neurogenic niche in the beginning plays a subordinate role. However, as the NBs in the proliferation zones disappear during early post-embryonic life, the neuronal precursors in the niche gradually become the dominant and only mechanism for the generation of new neurons in the adult brain.

## Materials and methods

### Animals

The marbled crayfish is a recently discovered 'parthenogenetic form' of the freshwater decapod *Procambarus fallax *[71]. Once the eggs are deposited by the female, embryonic development takes 22 to 42 days [39]. Embryos hatch to the first post-embryonic stage (POI) [39,48] and have well developed eyes, a full set of segments and appendage anlagen, and they are still attached to the mother's pleopods by a ligament coming from the telson. After 1 to 3 days, the POI stage molts into the post-embryonic stage II (POII) a free animal that still clings to the mother's pleopods. Like POI, POII still relies on a yolk supply stored under the dorsal carapace. After a second molt, animals become miniatures of the adult crayfish form (early juvenile, ADI), able to seek food independently but still looking for protection under the mother's pleon [39]. Although the animal is characterized by a genetically uniform progeny, its development is more or less identical to that of other crayfish. For these reasons, it is considered a suitable model organism for comparative developmental studies [37,38,40,41,72-84].

In the present study, marbled crayfish embryos and post-embryonic stages were taken from a laboratory culture in which single adult individuals (or one adult together with the juvenile progeny) were kept in small aquaria filled with oxygenated tap water (approximately 5 liters at 20°C) and a 10-h light/14-h dark regime. Aquaria were provided with pottery shelters, dried leaves and sand. Water was changed twice a week, and animals were normally fed three times a week with crustacean food pellets (TetraMin) and vegetables (carrots and peas) and checked for mortality, molts or freshly laid eggs. For the experiments, embryos were staged according to the developmental staging system established by Seitz *et al*. [39], which quantifies the percentage of developmental time from egg deposition to hatching.

### Standard immunohistochemical protocol

Crayfish embryos of different developmental stages were dissected in crayfish saline (mM: 205 NaCl, 5.4 KCl, 34.4 CaCl_2_, 1.2 MgCl_2_, 2.4 NaHCO_3_, pH 7.4). Brains were removed from the animals and either fixed for 4 h at room temperature (RT) or overnight at 4°C in 4% paraformaldehyde (PFA) in 0.1 M phosphate buffer (PB). Subsequently, brains were rinsed for 2 h in 0.1 M phosphate-buffered saline (PBS; pH 7.4), and incubated for half an hour in PBS plus 0.3% Triton X-100 (PBS-TX), before exposure to primary antibodies (4 h or overnight at 4°C). Specimens were then rinsed for 2 h in PBS, followed by 30 minutes in PBS-TX and then incubated overnight in the appropriate secondary antibodies. Some brains were also counterstained with the nuclear stain YOYO (0.25 μl/ml) for 15 minutes. Afterwards, samples were rinsed for 2 h in PBS and mounted either in Gelmount (Biomeda, Foster City, CA, USA) or Mowiol (coverslip mounting solution of polyvinyl alcohol (Mowiol 4.88, Calbiochem, San Diego, CA, USA; catalog number 475904).

### Antibody characterization

#### Monoclonal mouse anti-glutamine synthetase antibody

Monoclonal mouse anti-glutamine synthetase antibody (1:100) was from BD Biosciences Pharmingen (**Becton Dickinson, San Diego, CA**, USA. Number 610517). Glutamine synthetase is an octamer of 45 kDa subunits and catalyzes the amination of glutamic acid to form glutamine. The presence of two isoforms of glutamine synthetase have been found in the nervous system of *P. clarkii*, where enzymatic activity has been demonstrated [85]. In the brain of the spiny lobster *P. argus*, it was confirmed that glutamine synthetase immunostaining in the nervous system is specific for glia [33]. Antibodies against the sheep form of glutamine synthetase have been used in the present study to label cells in the developing neurogenic niche.

#### Monoclonal anti-tyrosinated tubulin

Monoclonal anti-tyrosinated tubulin (1:1,000) was from Sigma (**St. Louis, MO, USA**. T9028). Microtubules are mainly formed by tubulin, which is a heterodimer consisting of α-tubulin and ß-tubulin, subject to specific post-translational modifications at the carboxyl termini of both units [86]. One of these modifications involves the cyclic removal of the carboxy-terminal tyrosine of α-tubulin by a carboxypeptidase and the re-addition of a tyrosine residue by the tubulin-tyrosine-ligase. Evidence indicates that all interphase microtubules are initially tyrosinated and that daughter cells, immediately after mitosis, have primarily tyrosinated microtubules [87]. The monoclonal anti-tyrosine tubulin antiserum that we used reacts with tubulin's carboxy-terminal tyrosine (see product specifications). In the present study, this antibody has been used for localizing the tyrosinated α-tubulin in the brain and visualizing the main structure of the niche and the streams, which are rich in tubulin.

### S-phase markers

BrdU and EdU, synthetic thymidine analogs that are incorporated into single-stranded DNA during S-phase, can be used to identify cells actively in the cell cycle. Single and multiple labelings were performed and thus different cocktails of secondary antibodies were used. In single peroxidase BrdU labeling, the Cell Proliferation Kit RPN 20 (Amersham Int., Little Chalfont, Buckinghamshire, UK) was employed. The primary antibody was a mouse anti-BrdU included in the kit (1:100; Amersham, Cell Proliferation Kit RPN); details of the protocol are described below. For fluorescent staining, both the primary mouse anti-BrdU (Amersham kit) and the rat anti-BrdU (Sigma) were utilized. In this case, according to the goals of the experiment, goat anti-mouse Alexa 488 or 546, or goat anti-rat Alexa 488 or 546 (1:50; Invitrogen **Carlsbad, CA, USA**) was used. EdU, another modified nucleoside, was detected with a cell proliferation kit (Click-iT^® ^EdU Cell Proliferation Assays, Molecular probes **} Invitrogen™ Life Technologies, U.S.A**). EdU was detected only in fluorescently labeled preparations and always with the secondary Alexa 488 antibody, included in the kit. Details of the protocol are reported below.

#### Protocols for single labeling for BrdU or Edu

##### BrdU

Live juvenile crayfish or live hatchlings (after removal of the chorion) were incubated directly in a BrdU/saline solution (0.2 mg BrdU/ml; Cell Proliferation Kit RPN 20) for 4 h at RT. The 4-h time span has been demonstrated to provide an optimal labeling of cycling cells while the number of labeled cells is still low enough to be analyzed efficiently [2].

Subsequently, animals were fixed in PFA for 30 minutes at RT before dissection. Brains were removed and incubated in 4% PFA for at least 4 h at room temperature or overnight at 4°C. Specimens were washed in at least three changes of PBS buffer for 30 minutes and then pre-incubated in PBS-TX for 30 minutes. To denature the DNA and hence improve binding of the antibody, the tissue was exposed to an acid treatment (2N HCl) for 20 minutes. After another cycle of PBS rinses, brains were incubated in mouse anti-BrdU antiserum (1:100; Amersham, Cell Proliferation Kit RPN) for 2.5 h at RT. Afterwards, specimens were washed in PBS overnight at 4°C and incubated in goat anti-mouse antiserum conjugated with peroxidase for 60 to 90 minutes at RT. Specimens were washed in PBS overnight at 4°C. To detect the bound antibody, the enzymatic diaminobenzidine reaction was used according to the manufacturer's instructions: in the presence of cobalt and nickel, diaminobenzidine gives a blue-black precipitation where the secondary antibody is bound and hence in those nuclei that incorporated BrdU. After dehydration in a graded EtOH series, specimens were mounted in methyl salicylate.

##### EdU

The Click-iT^® ^EdU Cell Proliferation Assay (Molecular Probes) is an alternative to the BrdU method. It is not antibody-based and does not require DNA denaturation for detection of the incorporated nucleoside. Animals (embryos, hatchlings and juveniles) were incubated for 4 h at RT and afterwards fixed in PFA for 30 minutes. Brains were dissected and fixed for an additional 4 h. Specimens were then washed in at least three changes of PBS buffer and pre-treated in PBS-TX for 30 minutes. Incubation in Alexa 488 Click-iT reaction cocktail was performed for 2 h at RT. The solution was prepared fresh and contained 1X reaction buffer (CuSO_4_, AlexaFluor azide and reaction buffer additive). Several washing steps followed this application before the incubation in primary antibodies used for the double labeling.

#### BrdU-EdU pulse-chase experiment

In order to clarify the activity over time of cells residing in the niche, a series of pulse-chase experiments was performed. Animals, placed in a small container for liquid (100 ml volume), were exposed first to BrdU for 4 h (first pulse). Then, the labeling solution was quickly rinsed off the animals and these were kept undisturbed in fresh water for 24 h, 48 h or 7 days, depending on the experiment. During this time, animals were in similar conditions as before the treatment (that is, they were free to move, to interact with each other and to eat). After this survival period, animals were exposed to the second labeling reagent (EdU) for 4 h (second pulse) and immediately fixed and dissected. Protocols followed the directions described above and used rat anti-BrdU (1:100 in PBS-TX) and mouse anti-tyrosin-tubulin (1:1,000) antibodies overnight. The application of the Alexa 488 Click-iT reaction cocktail was performed for 2 h at RT. Tissues were then incubated for 4 h (or overnight at 4°C) in a cocktail of secondary antibodies (anti-mouse Alexa 630 and anti-rat Alexa 546). After final washings, brains were mounted in Mowiol mounting medium.

### Counterstaining

Actin and nuclear counterstaining provided more details of brain structure. Phalloidin-labeled phallo-toxins such as Alexa 488-phalloidin (1:50; N. A12379, Invitrogen) and Alexa 546 phalloidin (1:50; N. A12380, Invitrogen) are convenient probes for labeling and identifying F-actin in tissue. Using phalloidin, emerging structures of the brain during embryogenesis were highlighted, which allowed us to determine the exact developmental stages of specimens. As a nucleic acid stain, the green fluorescent YOYO (0.25 μl/ml; Invitrogen, NoY3601) was used in order to distinguish nuclei (hence cells) from acellular structures in the brain (for example, the central cavity or pore in the niche).

### Microscopic analysis

Digital images of light microscopic specimens were obtained with a Zeiss Axioskop fitted with a CCD-1300B digital camera (Vosskühler GmbH, Osnabrück, Germany) and processed with the Lucia Measurement 5.0 software package (Laboratory Imaging Ltd, Praha, Czech Republic)). Fluorescent samples were screened with a Zeiss LSM 510 Meta confocal microscope. Images are based on stacks of 15 to 20 optical sections (single images are averages of 4 to 8 laser sweeps) of a z-series. Measurement of cells residing in the niche were obtained using the software ImageJ. The size of images in two dimensions was 1,024 × 1,024 × 1 pixel; sizes of images in three dimensions (z stack) varied according to the sample. Images from stacks represent projection of the z sections. Some images were adjusted for brightness and contrast with Adobe Photoshop 7.0 software (Adobe Systems, CA, USA) and arranged into plates by using the illustration program Illustrator 11.0 (Adobe Systems).

### Injection of fluorescently labeled dextran into the embryonic crayfish (*P. clarkii*) brain

*P. clarkii *embryos just prior to hatching (E95% to 100%, n = 80) [48] were removed from an egg-bearing female. A small tear was made in the chorion of each egg to allow penetration of BrdU (Sigma; 2 mg/ml pond water), in which the embryos were incubated for 4 h. Individual eggs were then injected with dextran conjugated to fluorescent dye by penetrating the egg membranes and the body wall with a fine glass micropipette connected to a microinjection apparatus (Sutter Instruments, Novato, CA, USA). Dextran solution (0.26 μl of 2 mM dextran in saline; D-3306, anionic, lysine fixable MW 3000; Invitrogen) was injected into the dorsal hemolymph sinus of each embryo; embryos were maintained in pond water for 20 minutes after injection. Embryos were fixed for a minimum of 6 h, then dissected and brains were fixed for a total of 24 h with 4% PFA in 0.1 M phosphate buffer at 4°C. Brains were processed immunocytochemically for BrdU and tyrosinated tubulin as described above, with the exception that BrdU was detected using rat anti-BrdU (1:50; Accurate Chemical Company, Westbury, NY, USA) and goat anti-rat IgG conjugated to CY2 (Jackson ImmunoResearch Laboratories, Inc., West Grove, PA, USA). Brains were mounted in Fluoro-Gel, and examined and imaged using a Leica TCS SP5 confocal microscope equipped with argon 488 nm, and 561 and 633 nm diode lasers. Serial optical sections were taken at 1-μm intervals and saved as both three-dimensional stacks and two-dimensional projections.

## Abbreviations

ADI: first immature adult stage; BrdU: 5-bromo-2'-deoxyuridine; DPS: deutocerebral proliferative system; EdU: 5-ethynyl-2'-deoxyuridine; GMC: ganglion mother cell; LPZ: lateral proliferative zone; MPZ: medial proliferative zone; NB: neuroblast; PBS: phosphate buffer; PBS-TX: PBS plus 0.3% Triton X-100; PFA: paraformaldehyde; POI: first post-embryonic stage; POII: second post-embryonic stage; RT: room temperature.

## Competing interests

The authors declare that they have no competing interests related to this study.

## Authors' contributions

SS performed the primary experimental work and analysis for this paper, which contributed to her PhD thesis. JLB repeated aspects of this work in marbled crayfish, confirming the major findings of the study, and did the pulse-chase experiments with 1 week survival time. She also did the dextran injections of the vasculature in *P. clarkii*. BSB and SH contributed to the planning and analysis of experiments, as well as to the composition of the final manuscript. BSH assisted in drafting the manuscript. All authors read and approved the final manuscript.

## Supplementary Material

Additional file 1 **A video taken from a confocal stack**. The video from a confocal stack (Figure 13Aii) was imaged from the ventral surface through to the mid-brain of a *P. clarkii *brain at hatching (approximately 26 μm into the brain tissue). There are three labels: (1) dextran dye (green) micro-injected into the cerebral artery via the dorsal sinus combined with (2) the S-phase marker BrdU (red) identifying the cap and the immunolocalization of (3) tyrosinated tubulin (white) delineating the protoniche and its pore. The video shows that the cerebral artery bifurcates and then pushes ventrally toward the two hemispheres, each branch dividing laterally and then many times over into the neuropile regions as continuous tubes. The protoniche has blood vessels confluent with its structure and connects to blood vessels slightly deeper into the tissue. The central pore, considered as an early stage of the cavity, is confluent with the blood vasculature. Click here for file
